# The Therapeutic Efficacy of Danhong Injection Combined With Percutaneous Coronary Intervention in Acute Coronary Syndrome: A Systematic Review and Meta-Analysis

**DOI:** 10.3389/fphar.2018.00550

**Published:** 2018-06-04

**Authors:** Jun-Bo Zou, Xiao-Fei Zhang, Jing Wang, Fang Wang, Jiang-Xue Cheng, Fang-Yan Yang, Xiao Song, Yu Wang, Yu-Lin Liang, Ya-Jun Shi

**Affiliations:** ^1^College of Pharmacy, Shaanxi University of Chinese Medicine, Xianyang, China; ^2^Key Laboratory of Modern Preparation of Traditional Chinese Medicine, Ministry of Education, Jiangxi University of Traditional Chinese Medicine, Nanchang, China

**Keywords:** Danhong injection, percutaneous coronary intervention, acute coronary syndrome, systematic review, meta-analysis

## Abstract

**Background:** Percutaneous coronary intervention (PCI) is widely used in treatment of acute coronary syndrome (ACS) clinically. It is believed that Danhong injection (DHI) extracted from *salviae miltiorrhizae* and *flos carthami* combined with PCI could increase the therapeutic efficacy on ACS. We provide an updated meta-analysis with detailed information on combination of DHI and PCI therapy.

**Materials and Methods:** Electronic databases were searched for appropriate articles without language limitations on key words before October 22, 2017. All trails were screened according to certain criteria. Quality of eligible studies was also assessed. We made a detailed record of outcome measurements. RevMan 5.3 software was used to perform the meta-analysis.

**Results:** 14 articles involving 1533 patients with ACS were selected. Compared to PCI treatment alone, total efficacy rate (TER) was enhanced and major adverse cardiovascular events (MACE) were reduced significantly for the combination of DHI and PCI (*P* < 0.00001). Vascular endothelial function was improved by significantly decreasing the contents of ET-1, vWF and increasing the levels of NO and FMD (*P* < 0.00001). The serum levels of IL-1, IL-6, IL-18, TNF-α, LpPLA2, MMP-9, and pentraxin-3 were significantly decreased (*P* < 0.00001), whereas IL-10 in serum was increased (*P* < 0.00001), indicating a stronger anti-inflammatory effect of the combination. The combination therapy decreased the serum levels of CD62P, PAGT, PADT, FIB-C significantly (*P* < 0.05), which was beneficial for preventing coagulation of platelets. Blood lipid was also affected by regulating TC, TG, LDL, and HDL, but the results were not statistically significant (*P* > 0.05). Cardiac function was improved by increasing LEVF (*P* = 0.006) but not LVED (*P* = 0.08). The combination treatment was associated with an improvement in antioxidant effect by decreasing MDA and increasing SOD significantly (*P* < 0.00001).

**Conclusion:** Combination of DHI and PCI in treatment of ACS could improve TER and reduce incidence of MACE after PCI therapy. These effects may be mediated by combined actions of several mechanisms. However, these results of this study should be handled cautiously due to the limitations of this research. Several rigorous RCTs are in need to confirm these findings.

## Introduction

Acute coronary syndrome (ACS) is the most severe form of Cardiovascular disease (CVD) and CVD accounts for approximately one-third of all global deaths (Chu et al., 2017). Percutaneous coronary intervention (PCI) has become the most effective treatment for ACS (Sun et al., 2017). Despite reestablishing the epicardial coronary vessel patently, PCI may associate with some pathological mechanisms including vascular endothelial dysfunction, aggregation of platelets, diffuse myocardial edema, and neutrophilic plugging which would lead to some major adverse cardiac events (MACE) and/or poor prognosis (Movahed and Butman, 2008; Kirtane et al., 2014; Bouleti et al., 2015).

**FIGURE 1:**
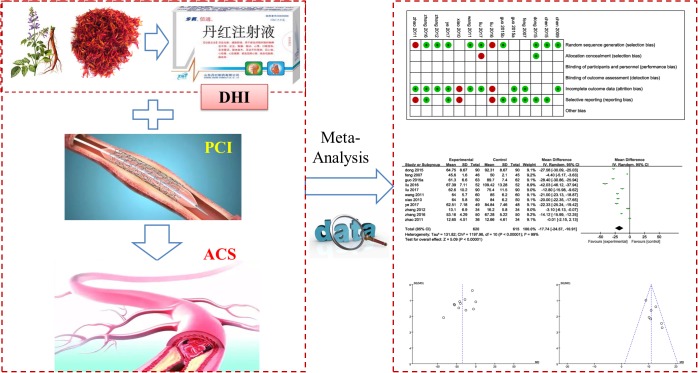
Work flow of present study.

Danhong injection (DHI) is a Chinese patent medicine extracted from *salviae miltiorrhizae* and *flos carthami*. DHI was approved by China Food and Drug Administration (CFDA) in 2002. Its major function is promoting blood circulation to remove blood stasis and dredging meridians. In the theory of TCM, the mechanism of ACS belongs to stagnant blood block and DHI serves to invigorate blood circulation and eliminate stasis which was proven to be efficacious in treating ACS (Wu et al., 2015). Modern pharmacological researches also have shown that DHI can improve the coronary circulation, reduce blood viscosity, and scavenge free radicals to control the occurrence of angina pectoris (Chen and Wang, 2011; Wu et al., 2015). DHI is believed to have obvious therapeutic effects for patients with CVD including coronary heart disease (CHD), angina, myocardial infarction (MI), and cerebral infarction (CI) (Wang et al., 2014). Several recent studies have found that DHI is beneficial to patients with ACS after PCI (Dong et al., 2014; Wu et al., 2015; Zheng et al., 2015; Xie et al., 2016; Zhang Z.H. et al., 2016). A newest meta-analysis reported DHI combined with PCI was superior to PCI therapy alone in treatment for periprocedural myocardial injury (Zhang et al., 2017). But the outcome measures in this paper were not comprehensive enough. Therefore, we provide an updated and extended meta-analysis with detailed information for the combination of DHI and PCI on patients with ACS (**Figure 1**).

## Methods and Program

### Literature Retrieval Strategy

Keywords “Danhong injection (DHI) or Danhong” [Title/Abstract] AND “percutaneous coronary intervention (PCI)” [Title/Abstract] AND “acute coronary syndrome (ACS)” [Title/Abstract] were used as search items in electronic databases including Pubmed, Wanfang, the China National Knowledge Infrastructure (CNKI), the VIP medicine information system (VMIS), Embase, the Cochrane Library and the Chinese Biomedical Database (CBM). Articles published before October 22, 2017 was examined without language limitations in order to obtain a comprehensive retrieval. All relevant articles were downloaded into Endnote software (version X7, Thomson Reuters, Inc., New York, NY, United States) for further exploring. Duplicate records were removed. Full-text review was performed while the title/abstract thought to be thematic. The job above was executed by two investigators independently. Conflicts were resolved by the consensus and discussion.

### Inclusion and Exclusion Criteria

According to the suggestions of a cardiologist, we designed the inclusion criteria as follows: (1) Patients in RCTs were diagnosed with ACS by meeting the criteria of Diagnostic Criteria of European Society of Cardiology Congress (DCESCC) version 2000, or Diagnosis and treatment of unstable angina pectoris(DTUAP) version 2000, or Diagnostic Criteria of World Health Organization (DTWHO) version 1981, or Clinical guideline of new drugs for traditional Chinese medicine(CGNDTCM), or Diagnostic Criteria of American College of Cardiology (DCACC) version 2007, or guidelines for diagnosis and treatment of acute myocardial infarction (GDTAMI) version 2001, or guidelines for diagnosis and treatment of coronary heart disease (GDTCHD) version 2007, or carrying out coronary arteriography or echocardiogram. (2) All trails mentioned were described as RCTs. (3) Patients in experimental group received PCI-based therapy with DHI, whereas patients in control group received PCI therapy only. (4) Outcome measurements of each study must have included a minimum of two of the following indices: P-selectin CD62(CD62P), fibrinogen C(FIB-C), high-sensitivity C-reactive protein(hsCRP), total cholesterol (TC), triglyceride(TG), low density lipoprotein (LDL), high density lipoprotein (HDL), left ventricular end-diastolic volume (LVED/LVEDV), left ventricular end-systolic volume (LVESV), left ventricular fraction (LVEF), Platelet aggregation (PAGT), Platelet Adhesion rate (PADT), major adverse cardiovascular events (MACE, mainly included MI, angina, sudden cardiac deaths and heart failure), ST segment resolution (STR), total Efficacy Rate (TER), endothelin-1 (ET-1), fibrinogen (Fg), von Willebrand factor (vWF), nitric oxide (NO), flow-mediated dilation (FMD), tumor necrosis factor-α (TNF-α), interleukin (IL), intercellular adhesion molecule-1 (ICAM-1), vascular cell adhesion molecule-1 (VCAM-1), brain natriuretic peptide (BNP), lipoprotein-associated phospholipase A2 (LpPLA2), matrix metalloproteinase (MMP).

An exclusion criterion was designed as follows: (1) Articles such as reviews, animal experiments, case report and comments et al. were thought to be unrelated with the topic. (2) Trails were not RCTs or Diagnostic criteria in statement were ambiguous. (3) The intervention of ACS patients was not based on PCI treatment.

### Data Extraction and Quality Assessment

Information of eligible studies including authors, year of publication, sample size, interventions and outcome measurements et al. were extracted and arranged to tables. Quality of included studies was assessed by two investigators independently according to the Cochrane Handbook for Systematic Reviews of Interventions (Deeks et al., 2011). Disagreement was resolved by the consensus. Quality assessment was evaluated as follows: random sequence generation (selection bias), allocation concealment (selection bias), blinding of participants and personnel (performance bias), blinding of outcome assessment (detection bias), incomplete outcome data (attrition bias), selective reporting (reporting bias) and other bias. Each term was judged with three levels. “Low risk” of bias means the description of methods or procedures was adequate, “High risk” indicates the description of methods or procedures was not adequate or incorrect while “Unclear risk” of bias means there was no description of methods and/or procedures.

### Data Analysis

Data analysis was performed using Review Manager 5.3 (Cochrane Collaboration). Outcome measures such as TER and MACE were regarded as dichotomous variables and presented as the odds ratio (OR) with 95% confidence intervals (95% CI), Contents of inflammatory cytokines (IL-1, IL-6, IL-10, IL-18, TNF-α, LpPLA2, MMP-9, and pentraxin-3), indices of platelet (CD62P, sP-sel, PAGT, PADT, and FIB-C) and factors of blood lipid (TC, TG, LDL, and HDL) et al. were continuous variables that presented as the mean difference (MD) with 95% CI. *Q* statistic and *I*^2^ tests were applied to assess the heterogeneity among studies. A fixed-effects model was used to analyze data with low heterogeneity (*P* ≥ 0.1 and *I*^2^ ≤ 50%) and data with high heterogeneity (*P* < 0.1 or *I*^2^ > 50%) was estimated using random-effects model. Potential publication bias was revealed by funnel plots.

## Results

### Characteristics of the Eligible Studies

**FIGURE 2:**
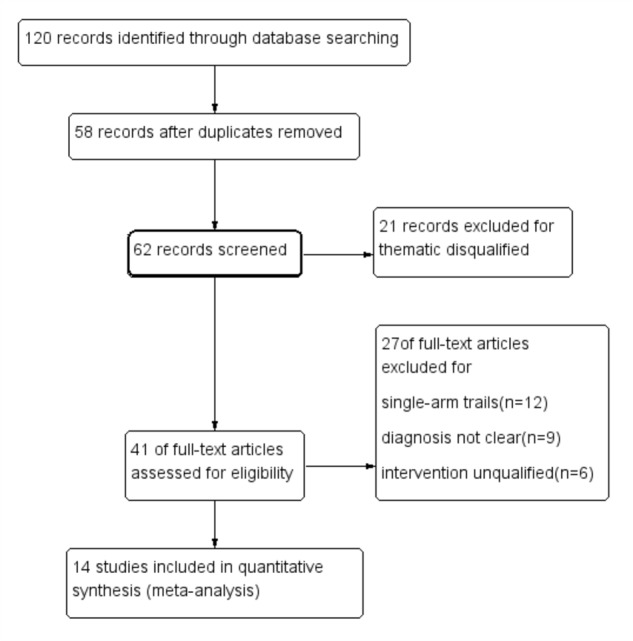
Process of study extracted for the meta-analysis.

One hundred and twenty articles were identified through database searching, in which 58 articles were removed for duplicates. 21 articles in 62 remaining were excluded for thematic disqualification. Then, 41 articles remained for further full-text review. 21 studies were excluded in this procedure for the following reasons: Diagnosis in 9 articles was vague, 6 studies mentioned unfit interventions and 12 studies were single-arm designs. 14 studies (Feng et al., 2007; Chen et al., 2009, 2015; Xiao and Cao, 2010; Wang et al., 2011; Zhao and Jiang, 2011; Zhang and Zhang, 2012; Dong and Du, 2015; Guo J.F. et al., 2015; Guo L.L. et al., 2015; Liu et al., 2016, 2017; Zhang W.W. et al., 2016; Ye et al., 2017) were included in quantitative synthesis finally (**Figure 2**).

One thousand, five hundred and thirty-three patients diagnosed with ACS (769 cases in the experimental group and 764 cases in the control group) were taken in this meta-analysis. The age of the patients ranged from 45 to 87 years, and there was no obvious difference in terms of age and sex between the two groups (**Table 1**). Trails were conducted between 2007 and 2017; all were RCTs with a comparison between a combination of DHI and PCI therapy and PCI therapy only. The PCI therapy in eligible trails varied slightly and the usual regimen was the combination of clopidogrel, aspirin, low molecular heparin (LMH), β-receptor blocker or ACEI in PCI treatment. The dose of DHI ranged from 20 to 40 mL/day via intravenous drip. Thirteen studies reported the duration of treatment lasted for 2 weeks. Four trails reported a follow up ranged from 2 to 6 months (**Table 2**).

**Table 1:** Characteristics of included studies.

Author, year	Cases T/C	Diagnostic standard	Age (years) Range, mean		Sex Male/female	
Chen et al., 2009	50/50	DCESCC(2000)&CA	T:50–72, 63.1	C: 51–74,67.5	T: 32/18	C: 30/20
Chen et al., 2015	60/60	EC	T:61.4	C: 61.5	T: 36/24	C: 39/21
Dong and Du, 2015	90/90	DTUAP(2000)	T: 45–76, 62.4	C: 46–78, 62.5	T: 48/42	C: 54/36
Feng et al., 2007	45/46	CA	T: 67.2	C: 65.6	T: 34/12	C: 32/13
Guo et al., 2015	63/62	DCWHO(1981)& CGNDTCM	T: 55–79, 62.1	C: 53–76, 61.5	T: 33/30	C: 36/26
Guo et al., 2015	40/38	NR	T:61.6	C: 60.1	T: 23/17	C: 22/16
Liu et al., 2016	52/52	EC	T: 47–73, 58.5	C: 48–72, 59.2	T: 28/24	C: 27/25
Liu et al., 2017	90/90	GDTAMI(2001)	NR	NR	NR	NR
Wang et al., 2011	60/60	DCESCC(2000)&CA	T: 56–79, 67.8	C: 54–76, 65.8	T: 31/29	C: 28/32
Xiao and Cao, 2010	50/50	DCESCC(2000)&CA	T: 56–79, 67.8	C: 54–76, 65.8	T: 26/24	C: 27/23
Ye et al., 2017	49/48	DCWHO(1981)	T: 52–87, 64.1	C: 51–86, 64.3	T: 28/21	C: 26/22
Zhang and Zhang, 2012	34/34	DCACC(2007)&CA	T: 55.7	C: 54.5	T: 18/16	C: 19/15
Zhang et al., 2016	50/50	GDTCHD(2007)	T: 61–80, 71.3	C: 61–79, 68.3	T: 33/17	C: 34/16
Zhao and Jiang, 2011	36/34	DCACC(2007)	T: 54.0	C: 54.0	T: 19/17	C: 18/16

*T, trial group; C, control group; NR, no report. DCESCC, Diagnostic Criteria of European Society of Cardiology Congress; CA, coronary arteriography; EC, echocardiogram; DTUAP, diagnosis and treatment of unstable angina pectoris; DCWHO, Diagnostic Criteria of World Health Organization; CGNDTCM, clinical guideline of new drugs for traditional Chinese medicine; DCACC, Diagnostic Criteria of American College of Cardiology; GDTCHD, guidelines for diagnosis and treatment of coronary heart disease; GDTAMI, guideline for diagnosis and treatment of acute myocardial infarction.*

**Table 2:** Intervention characteristics of included studies.

Study ID (name, year)	Essential treatment and drugs for ACS	DHI dosage and method	Duration/follow up	Outcome measures
Chen et al., 2009	PCI + Clopidogrel + Aspirin et al.	40 mL/day, intravenous drip	2 weeks/NR	CD62P, GP IIb/IIIa, FIB-C, hs-CRP
Chen et al., 2015	PCI + Clopidogrel + Aspirin et al.	40 mL/day, intravenous drip	2 weeks/6 months	TC, TG, LDL, HDL, LVED, LVEF, PAGT, PADT, CD62P, MACE
Dong and Du, 2015	PCI + Clopidogrel + Aspirin + LMH et al.	20 mL/day, intravenous drip	2 weeks/NR	TER, vWF, ET-1, NO, FMD, SOD, MDA
Feng et al., 2007	PCI + Clopidogrel + Aspirin et al.	40 mL/day, intravenous drip	4 weeks/NR	TC, TG, LDL, HDL, hs-CRP, ET, Fg, MACE
Guo et al., 2015	PCI + Clopidogrel + Aspirin et al.	40 mL/day, intravenous drip	2 weeks/2 months	vWF, ET-1, NO, FMD, TNF-α, IL-1, CRP, MACE
Guo et al., 2015	PCI + Clopidogrel + Aspirin + LMH et al.	40 mL/day, intravenous drip	14 days/NR	hs-CRP, ICAM-1, VCAM-1
Liu et al., 2016	PCI + Clopidogrel + Aspirin + LMH et al.	40 mL/day, intravenous drip	2 weeks/NR	vWF, ET-1, NO, FMD, NTG, IL-18, IL-10, LpPLA2, LVESV, LVEDV, LVEF, BNP, pentraxin-3
Liu et al., 2017	PCI + Clopidogrel + Aspirin + LMH et al.	20 mL/day, intravenous drip	14 days/NR	TER, hsCRP, ET, LVEF, LVEDV
Wang et al., 2011	PCI + Clopidogrel + Aspirin + LMH et al.	40 mL/day, intravenous drip	2 weeks/NR	vWF, ET-1, NO
Xiao and Cao, 2010	PCI + Clopidogrel + Aspirin et al.	20 mL/day, intravenous drip	14 days/NR	vWF, ET-1, NO, FMD
Ye et al., 2017	PCI + Clopidogrel + Aspirin et al.	40 mL/day, intravenous drip	3 months/6 months	TER, ET-1, NO, MACE
Zhang and Zhang, 2012	PCI + Clopidogrel + Aspirin + LMH et al.	40 mL/day, intravenous drip	14 days/NR	ET-1, CD62P, hs-CRP, STR
Zhang et al., 2016	PCI + Clopidogrel + Aspirin + LMH et al.	40 mL/day, intravenous drip	2 weeks/2 months	vWF, ET-1, NO, FMD, IL-6, MMP-9, hs-CRP
Zhao and Jiang, 2011	PCI + Clopidogrel + Aspirin + LMH et al.	40 mL/day, intravenous drip	2 weeks/NR	ET-1, sP-sel, hs-CRP, MACE

*ACS, acute coronary syndrome; PCI, percutaneous coronary intervention; DHI, Danhong injection; CD62P, P-selectin CD62P; GP, glucose protein; FIB-C, fibrinogen C; hs-CRP, high-sensitivity C-reactive protein; TC, total cholesterol; TG, triglyceride; LDL, low density lipoprotein; HDL, high density lipoprotein; LVED/LVEDV, left ventricular end-diastolic volume; LVESV, left ventricular end-systolic volume; LVEF, left ventricular fraction; PAGT, platelet aggregation; PADT, platelet adhesion rate; MI: myocardial infarction; MACE, major adverse cardiac events; LMH, low molecular heparin; MACE, major adverse cardiovascular events; STR, ST segment resolution; TER, total efficacy rate; ET-1, endothelin-1; Fg, fibrinogen; vWF, von Willebrand factor; NO, nitric oxide; FMD, flow-mediated dilation; TNF-α, tumor necrosis factor-α; IL, interleukin; ICAM-1, intercellular adhesion molecule-1; VCAM-1, vascular cell adhesion molecule-1; NTG, endothelial non-dependent vascular dilation reactions; BNP, brain natriuretic peptide; LpPLA2, lipoprotein-associated phospholipase A2; MMP, matrix metalloproteinase; sP-sel, soluble P-selectin; NR, no report.*

### Quality of Included Trials Assessment

**FIGURE 3:**
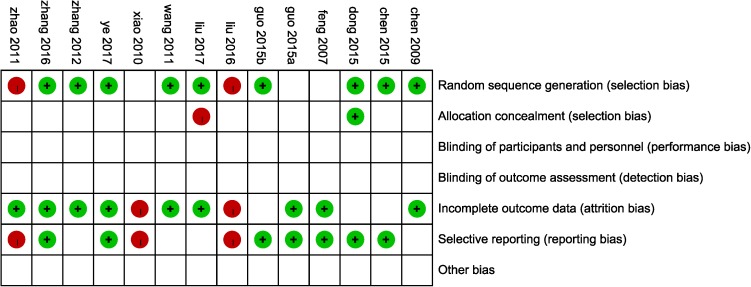
Risk of bias assessment in eligible studies. The quality assessment was conducted by Review Manager 5.3 according to Cochrane Handbook for Systematic Reviews of Interventions Version 5.1.0. Red circle, high risk of bias; green circle, low risk of bias; blank, unclear risk of bias.

According to the Cochrane risk of bias estimation, all trails mentioned a randomized allocation of participants while nine trails (Chen et al., 2009, 2015; Wang et al., 2011; Zhang and Zhang, 2012; Dong and Du, 2015; Guo L.L. et al., 2015; Zhang W.W. et al., 2016; Liu et al., 2017; Ye et al., 2017) described the appropriate generation of the random allocation sequence. Detailed information on allocation concealment of majority studies was missing. Blinding of participants and outcome assessment of all studies was not mentioned. Nine studies (Feng et al., 2007; Chen et al., 2009; Wang et al., 2011; Zhao and Jiang, 2011; Zhang and Zhang, 2012; Guo J.F. et al., 2015; Zhang W.W. et al., 2016; Liu et al., 2017; Ye et al., 2017) were at low risk of attrition bias for having been given a complete outcome data. Seven trails (Feng et al., 2007; Chen et al., 2015; Dong and Du, 2015; Guo J.F. et al., 2015; Guo L.L. et al., 2015; Zhang W.W. et al., 2016; Ye et al., 2017) reported the result of detailed indices indicated a low risk of reporting bias (**Figure 3**).

### Outcome Measures With Subgroup Analysis

#### TER and MACE of DHI Combined With PCI vs. PCI Therapy Alone

**FIGURE 4:**
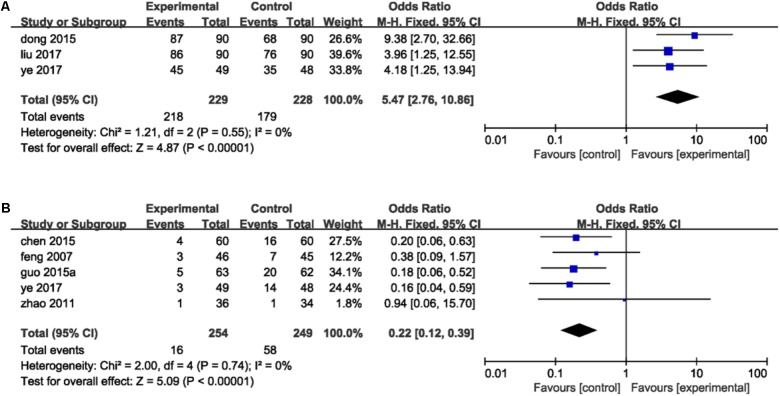
Forest plot of TER and MACE in patients treated with DHI + PCI and PCI alone. **(A)** The plot of TER, **(B)** the plot of MACE. *I*^2^ and *P* are the criterion for the heterogeneity test. ♦ Pooled odds ratio, —
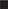
— odds ratio and 95%CI.

Criterion of TER was set as follows: ecovery was defined as the absence of symptoms while anginal attacks decreased significantly and duration of attacks decreased more than 80%. Effectiveness was identified that the symptoms were ameliorated and lower frequency of anginal attacks and duration decreased between 50 and 80%. Symptoms and anginal attacks remaining unchanged or worsening were defined as invalidation. TER refers to the proportion of patients who were evaluated to recovery and effectiveness in total groups. Three studies (Dong and Du, 2015; Liu et al., 2017; Ye et al., 2017) reported the total efficacy rate. A meta-analysis of these trails using a fixed-effect model demonstrated that DHI combined with PCI treatment significantly improved TER in the treatment of ACS (OR = 5.47, 95%CI: 2.76, 10.86; *P* < 0.00001). There was no statistically significant heterogeneity among individual trails (*P* = 0.55, *I*^2^ = 0%; **Figure 4A**). Five trails (Feng et al., 2007; Zhao and Jiang, 2011; Chen et al., 2015; Guo J.F. et al., 2015; Ye et al., 2017) provided descriptions on MACE after PCI therapy such as MI, angina, sudden cardiac deaths and heart failure. A fixed-effect model analysis certified that the combination of DHI and PCI treatment reduced the incidence of MACE significantly (OR = 0.22, 95%CI: 0.12, 0.39; *P* < 0.00001). No statistically significant heterogeneity was found among individual studies (*P* = 0.74, *I*^2^ = 0%; **Figure 4B**).

#### Indices of Vascular Endothelial Function of DHI Combined With PCI Therapy vs. PCI Therapy Alone

**FIGURE 5:**
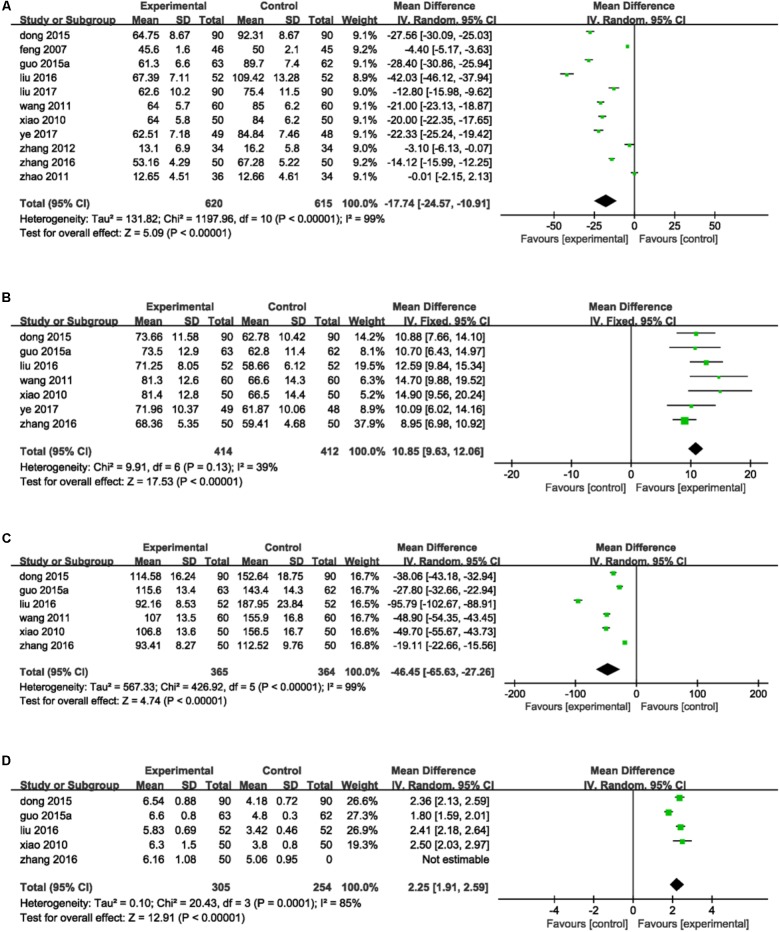
Forest plot of indices of vascular endothelial function in patients treated with DHI + PCI and PCI alone. **(A)** The plot of ET-1, **(B)** the plot of NO, **(C)** the plot of vWF, and **(D)** the plot of FMD. *I*^2^ and *P* are the criterion for the heterogeneity test. ♦ Pooled odds ratio, —
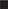
—mean difference and 95%CI.

ET-1, NO, vWF, and FMD were the main indices that mentioned in included studies reflected vascular endothelial function. Eleven studies (Feng et al., 2007; Xiao and Cao, 2010; Wang et al., 2011; Zhao and Jiang, 2011; Zhang and Zhang, 2012; Dong and Du, 2015; Guo J.F. et al., 2015; Liu et al., 2016, 2017; Zhang W.W. et al., 2016; Ye et al., 2017) reported the detection of ET-1. There was statistically significant heterogeneity among individual studies (*P* < 0.00001, *I*^2^ = 99%), so a random-effect model was applied to take a meta-analysis which demonstrated that the combination of DHI and PCI treatment significantly decreased the level of ET-1 in serum (MD = -17.74, 95%CI: -24.57, -10.91; *P* < 0.00001; **Figure 5A**). Seven trails (Xiao and Cao, 2010; Wang et al., 2011; Dong and Du, 2015; Guo J.F. et al., 2015; Liu et al., 2016; Zhang W.W. et al., 2016; Ye et al., 2017) provided the contents of NO. A fixed-effect analysis certified that DHI combined with PCI significantly increased the serum level of NO compared to PCI alone (MD = 10.85, 95%CI: 9.63, 12.06; *P* < 0.00001). No statistical significant was observed among individual studies (*P* = 0.13, *I*^2^ = 39%; **Figure 5B**). Detection of vWF was reported in six trails (Xiao and Cao, 2010; Wang et al., 2011; Dong and Du, 2015; Guo J.F. et al., 2015; Liu et al., 2016; Zhang W.W. et al., 2016). Heterogeneity was found among individual studies (*P* < 0.00001, *I*^2^ = 99%) and then a random-effect analysis was applied to demonstrate that DHI combined with PCI significantly decreased the serum content of vWF (MD = -46.45, 95%CI: -65.63, -27.26; *P* < 0.00001; **Figure 5C**). Five studies (Xiao and Cao, 2010; Dong and Du, 2015; Guo J.F. et al., 2015; Liu et al., 2016; Zhang W.W. et al., 2016) provided data of FMD. There was heterogeneity among individual trails (*P* = 0.0001, *I*^2^ = 85%) and a meta-analysis using a random-effect proved that combination of DHI and PCI could significantly increase the level of FMD of vascular endothelium (MD = 2.25, 95%CI: 1.91, 2.59; *P* < 0.00001; **Figure 5D**).

#### Inflammatory Indices of DHI Combined With PCI Therapy vs. PCI Therapy Alone

**FIGURE 6:**
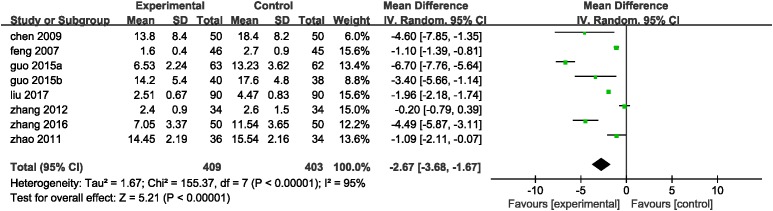
Forest plot of hsCRP in patients treated with DHI + PCI and PCI alone. *I*^2^ and *P* are the criterion for the heterogeneity test. ♦ Pooled odds ratio, —
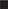
—mean difference and 95%CI.

Inflammation hypothesis of ACS proposed by Ross (1999) indicates that inflammation plays an important role in plaque buildup within the coronary arteries. Inflammatory indices reported in eligible studies including hsCRP, IL-1, IL-6, IL-10, IL-18, TNF-α, LpPLA2, MMP-9, and pentraxin-3. Eight trails (Feng et al., 2007; Chen et al., 2009; Zhao and Jiang, 2011; Zhang and Zhang, 2012; Guo J.F. et al., 2015; Guo L.L. et al., 2015; Zhang W.W. et al., 2016; Liu et al., 2017) mentioned the investigation on hsCRP. A random-effect model was used because of heterogeneity existence (*P* < 0.00001, *I*^2^ = 95%). A meta-analysis demonstrated that DHI combined with PCI significantly decreased the serum level of hsCRP (MD = -2.67, 95%CI: -3.68, -1.67; *P* < 0.00001; **Figure 6**). One trail (Guo J.F. et al., 2015) reported the serum IL-1 and TNF-α level. The serum levels of IL-10, IL-18, LpPLA2, and pentraxin-3 were measured in one study (Liu et al., 2016), and another one trail (Zhang W.W. et al., 2016) provided the IL-6 and MMP-9 level. The MD with 95%CI for IL-1, IL-6, IL-18, TNF-α, LpPLA2, MMP-9, and pentraxin-3 were (MD = -33.87, 95%CI: -37.45, -30.29), (MD = -14.41, 95%CI: -16.79, -12.03), (MD = -46.88, 95%CI: -60.46, -33.30), (MD = -27.79, 95%CI: -31.88, -23.70), (MD = -55.16, 95%CI: -60.00, -50.32), (MD = -107.93, 95%CI: -137.84, -78.02), and (MD = -2.41, 95%CI: -2.68, -2.14), respectively, indicating a significant decrease in the inflammatory indices in the experimental group compared with control group (*P* < 0.00001). The MD with 95%CI for IL-10 was (MD = 7.63, 95%CI: 5.82, 9.44) certifying a significant increase in the DHI + PCI group compared with PCI therapy alone (*P* < 0.00001; **Table 3**).

**Table 3:** Danhong injection (DHI) combined with PCI vs. essential treatment on inflammatory factors.

Inflammatory factors	Number of studies	Study ID	Cases of experimental group	Cases of control group	MD [95%CI]	*Z*-value	*P*-value
IL-1	1	Guo et al., 2015	63	62	-33.87 [-37.45, -30.29]	18.54	<0.00001
IL-6	1	Zhang et al., 2016	50	50	-14.41 [-16.79, -12.03]	11.89	<0.00001
IL-10	1	Liu et al., 2016	52	52	7.63 [5.82, 9.44]	8.26	<0.00001
IL-18	1	Liu et al., 2016	52	52	-46.88 [-60.46, -33.30]	6.77	<0.00001
TNF-α	1	Guo et al., 2015	63	62	-27.79 [-31.88, -23.70]	13.31	<0.00001
LpPLA2	1	Liu et al., 2016	52	52	-55.16 [-60.00, -50.32]	22.34	<0.00001
MMP-9	1	Zhang et al., 2016	50	50	-107.93 [-137.84, -78.02]	7.07	<0.00001
Pentraxin-3	1	Liu et al., 2016	52	52	-2.41 [-2.68, -2.14]	17.68	<0.00001

#### Platelet Activation Indices of DHI Combined With PCI Therapy vs. PCI Therapy Alone

CD62P, sP-sel, PAGT, PADT, and FIB-C were the indices of platelet activation recorded in eligible studies. Three trail (Chen et al., 2009, 2015; Zhang and Zhang, 2012) mentioned the determination of serum CD62P. One study (Zhao and Jiang, 2011) reported sP-sel, one trail (Chen et al., 2015) provided PAGT and PADT, and two trails (Feng et al., 2007; Chen et al., 2009) recorded FIB-C. The MD with 95% CI for CD62P, PAGT, PADT, and FIB-C were (MD = -1.82, 95%CI: -2.59, -1.06), (MD = -2.49, 95%CI: -3.73, -1.25), (MD = -1.07, 95%CI: -1.95, -0.19), and (MD = -1.50, 95%CI: -2.18, -0.83), respectively, indicating a significant decrease in experimental group in coagulation of platelet (*P* < 0.05). Compared to control group, sP-sel level of experimental group decreased slightly (MD = -0.80, 95%CI: -6.98, 5.38) but without statistical significance (*P* = 0.80; **Table 4**).

**Table 4:** Danhong injection combined with PCI vs. essential treatment on platelet activation.

Platelet activation indices	Number of studies	Study ID	Cases of experimental group	Cases of control group	MD [95%CI]	*Z*-value	*P*-value
CD62P	3	Chen et al., 2009, 2015; Zhang and Zhang, 2012	144	144	-1.82 [-2.59, -1.06]	4.66	<0.00001
sP-sel	1	Zhao and Jiang, 2011	36	34	-0.80 [-6.98, 5.38]	0.25	0.80
PAGT	1	Chen et al., 2015	60	60	-2.49 [-3.73, -1.25]	3.94	<0.0001
PADT	1	Chen et al., 2015	60	60	-1.07 [-1.95, -0.19]	2.40	0.02
FIB-C	2	Feng et al., 2007; Chen et al., 2009	96	95	-1.50 [-2.18, -0.83]	4.35	<0.0001

#### Influence on Blood Lipid of DHI Combined With PCI vs. PCI Alone

Overweight and dyslipidaemia were the 1st and 2nd prevalent factors leading to ACS (Vazquez-Arce and Marques-Sule, 2017). TC, TG, LDL, and HDL were highly related to overweight and dyslipidaemia which were reported in two trails (Feng et al., 2007; Chen et al., 2015). The MD with 95% CI for TC, LDL, and HDL were (MD = -0.11, 95%CI: -0.40, 0.18), (MD = -0.14, 95%CI: -0.38, 0.09) and (MD = -0.07, 95%CI: -0.32, 0.18), respectively, revealing a decrease in blood lipid but without statistical significance (*P* > 0.05). The MD with 95%CI (MD = 0.06, 95%CI: -0.14, 0.27) indicating a slightly increase in TG without significance (*P* > 0.05; **Table 5**).

**Table 5:** Danhong injection combined with PCI vs. essential treatment on blood lipid.

Indices of blood lipid	Number of Studies	Study ID	Cases of experimental group	Cases of control group	MD [95%CI]	*Z*-value	*P*-value
TC	2	Feng et al., 2007; Chen et al., 2015	105	106	-0.11 [-0.40, 0.18]	0.77	0.44
TG	2	Feng et al., 2007; Chen et al., 2015	105	106	0.06 [-0.14, 0.27]	0.63	0.53
LDL	2	Feng et al., 2007; Chen et al., 2015	105	106	-0.14 [-0.38, 0.09]	1.18	0.24
HDL	2	Feng et al., 2007; Chen et al., 2015	105	106	-0.07 [-0.32, 0.18]	0.57	0.57

#### Influence on Cardiac Function and Oxidation State of DHI Combined With PCI vs. PCI Alone

Three trails (Chen et al., 2015; Liu et al., 2016; Liu et al., 2017) reported the LVEF and LVED reflecting cardiac function. One trail (Dong and Du, 2015) provided measures of SOD and MDA in representation of oxidation state. There were heterogeneity in LVEF and LVED (*P* < 0.00001, *I*^2^= 93%; *P* < 0.0001, *I*^2^= 90%, respectively) and a random effect model was thus used for analysis. The MD and 95% CI for LVEF was (MD = 5.68, 95%CI: 1.61, 9.76), indicating a significant increase of LVEF in experimental group (*P* = 0.006). A meta-analysis demonstrated that DHI combined with PCI decreased LVED (MD = -5.89, 95%CI: -12.51, 0.74) non-significantly (*P* = 0.08). An analysis certified that the combination of DHI and PCI increased SOD level (MD = 22.17, 95%CI: 15.07, 29.27) and decreased MDA (MD = -1.09, 95%CI: -1.38, -0.80) significantly (*P* < 0.00001; **Table 6**).

**Table 6:** Danhong injection combined with PCI vs. essential treatment on cardiac function and oxidation state.

Item	Indices	Number of studies	Study ID	Cases of experimental group	Cases of control group	MD [95%CI]	*Z*-value	*P*-value
Cardiac function	LVEF	3	Chen et al., 2015; Liu et al., 2016, 2017	202	202	5.68 [1.61, 9.76]	2.73	0.006
	LVED	3	Chen et al., 2015; Liu et al., 2016, 2017	202	202	-5.89 [-12.51, 0.74]	1.74	0.08
Oxidation state	SOD	1	Dong and Du, 2015	90	90	22.17 [15.07, 29.27]	6.12	<0.00001
	MDA	1	Dong and Du, 2015	90	90	-1.09 [-1.38, -0.80]	7.37	<0.00001

#### Publication Bias

**FIGURE 7:**
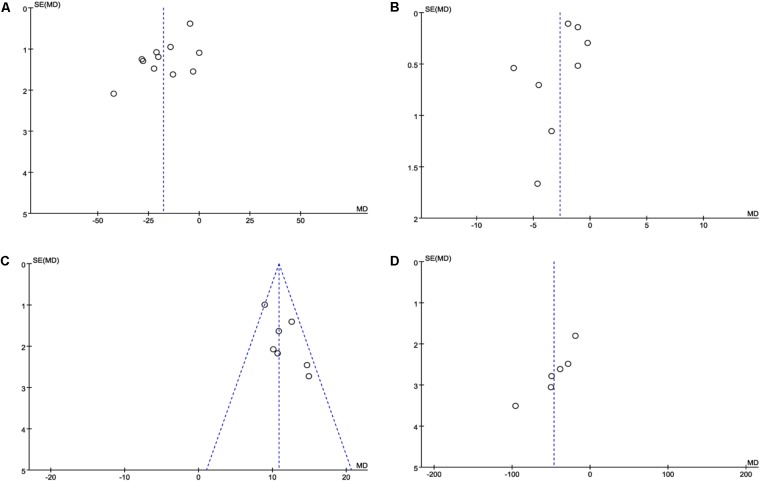
Funnel plot for the publication bias. **(A)** The plot of ET-1, **(B)** the plot of hsCRP, **(C)** the plot of NO, and **(D)** the plot of vWF. The funnel plots of these factors were symmetric, indicating that the publication was small.

Publication bias was expressed by a funnel plot. In this study, funnel plots of combination of DHI and PCI vs. PCI therapy alone on ET-1, hs-CRP, NO and vWF were applied. The plots were generally symmetric, suggesting that there was no obvious publication bias (**Figures 7A–D**).

## Discussion

Cardiovascular Disease (CVD) is the main cause of death which will be the first “killer” for human beings by 2020 (Lopez and Murray, 1998). CVD is the most common disease in China and ACS is the most severe form accompanied by highly disability rate, fatality rate and multi-complications which places gravely threat on human health (Zheng et al., 2014). Despite PCI is more effective in restoring coronary blood flow compared with other interventions, itself would be a dangerous factor that results in poor prognosis. MI associated with PCI now is a new type in the new classification for acute myocardial infarction (Makki et al., 2015). There are some management of PCI-related complications in patients who have undergone PCI. However, they usually bring some new risks such as bleeding (Hansen et al., 2010), intracranial hemorrhage (Hess et al., 2015), in-stent restenosis (Chen et al., 2015) and some other considerable harms (Cannon et al., 2017) which make PCI a difficult challenge in treatment of ACS for patients.

Though there are some limitations for TCM due to the lack of enough basic research, increasingly effective evidence-based practice makes it an eye-catching therapy system for many diseases. Danhong injection extracted from *salviae miltiorrhizae* and *flos carthami*, is a star representative in TCM for treating cardiovascular and cerebrovascular diseases. *Salviae miltiorrhizae* serves as monarch drug in the prescription, taking on effectiveness including antithrombus, improving microcirculation and antioxidant (Qi et al., 2017). *Flos carthami* plays a role of ministerial drug taking effects of improving hemorheology, inhibiting the aggregation of platelets, decreasing myocardial reperfusion and enhancing vascular endothelial function (Pei, 2005).

In clinical trials, a surrogate endpoint (or marker) is a measure of effect of a specific treatment that may correlate with a real clinical endpoint but does not necessarily have a guaranteed relationship. Interestingly, we find that surrogate endpoints were used in most of the analyzed trials (cytokines, endothelin etc…), while clinical endpoints were used only in some trials in this meta-analysis. The popularity of surrogate endpoints in ACS is for many reasons. Firstly, the primary clinical endpoints (such as MACE) may cause life threatening problems to patients, thus making it impractical to conduct a clinical trial to assess the antianginal effects of a drug. Secondly, surrogate endpoints selected in eligible trails could be easily measured prior to the occurrence of MACE and highly correlated with the process of ACS which often leads to dramatic reductions in sample size and much shorter studies than use of the clinical endpoints. What’s more important, the use of surrogate endpoints could speed up the arrival of scientific conclusion and is in favor of making early decisions on clinical protocols in ACS.

The endothelium plays an important role in the pathogenesis of ACS (Cieslik-Guerra et al., 2014), and circulating endothelial cells (CECs) have been put forward as a promising biomarker for diagnosis and prognosis of coronary artery disease and ACS (Schmidt et al., 2015). The increased ET-1 level in active coronary lesions may lead to vasospasm and to the progression of atherosclerosis (Ihling, 1998). NO maintains endothelial balance by controlling cellular processes of vascular smooth muscle cells. The variations in the NO pathway could include atherosclerotic events (Umman et al., 2015). High level of vWF is partly due to endothelial dysfunction and atherosclerosis, and it is associated with an increased risk of coronary artery disease (Sonneveld et al., 2015). FMD of the brachial artery has been recommended as non-invasive methods to assess endothelial structure and function (Kalay et al., 2010). Here we certified that the combination of DHI and PCI could improve the vascular endothelial function by decreasing the contents of ET-1 and vWF, increasing the levels of NO and FMD (*P* < 0.00001).

Inflammatory response has been considered as an important mechanism for ACS (Santos-Gallego et al., 2014). Thrombolytic therapy in essential treatment of PCI would up-regulate inflammatory mediators including hs-CRP and TNF-α compared with PCI alone, which suggests an increasing risk of detrimental effects on myocardium (Garjani et al., 2016). An increase of hs-CRP is a reflex of unstable plaque and poor prognosis (Ross, 1999). TNF-α is a pro-inflammatory cytokine that could be a potential biomarker in ACS due to its multiple functions (Sandoval-Pinto et al., 2016). Here we found that DHI combined with PCI could not only decrease the serum level of hs-CRP (MD = -2.67, 95%CI: -3.68, -1.67; *P* < 0.00001) and TNF-α (MD = -27.79, 95%CI: -31.88, -23.70; *P* < 0.00001), but also decrease other inflammatory factors including IL-1, IL-6, IL-18, LpPLA2, MMP-9, and pentraxin-3 significantly (*P* < 0.00001). IL-6 is an independent predictor of adverse events in low-moderate risk patients with NSTE-ACS and troponin-negative (Garcia-Salas et al., 2014). IL-18 level is a valuable parameter for risk of MACE in patients with ACS (Zhou et al., 2014). Lp-PLA_2_ levels are related to plaque stability (Chung et al., 2014) which represents the crossroad between lipid metabolism and inflammatory response (Abbate et al., 2012). Serum MMP-9 could be an early marker that discriminates MI from UA and predicts poor outcome in terms of disease severity and extent of disease complications (Hamed and Fattah, 2015). What’s more, the combination further enhanced anti-inflammatory effects via increasing serum level of IL-10 (MD = 7.63, 95%CI: 5.82, 9.44; *P* < 0.00001).

Antiplatelet agents form the cornerstone of medical therapy in patients with ACS (Makki et al., 2015). CD62P expressing functional thymic stromal lymphopoietin receptors (TSLPR) which promote platelet activation may be one of the mechanisms involved in thrombosis in ACS (Wang et al., 2013). PAGT and PADT play important parts in the occurrence and development of CHD as manifested by the enhancement of platelet’s adherence and aggregation (Jia, 1989). Increased FIB levels are independently associated with intermediate-high syntax score which is related to more serious disease and worse prognosis in patients with ACS (Kurtul et al., 2016). sP-sel is demonstrated to have prognostic values in predicting the cardiac events in patients with preserved left ventricular systolic function (Chen et al., 2013). We provided that DHI combined with PCI significantly decreased serum levels of CD62P, PAGT, PADT, and FIB-C (*P* < 0.05). The combination also decreased the level of sP-sel but without statistical significance (*P* = 0.80).

Hyperlipidemia is a major risk factor for CHD and potential benefit can be obtained by early treatment of hyperlipidemia following ACS (Balci, 2011). A series of changes in TC, TG, LDL, and HDL occur during acute phase response. However, DHI did not influence the serum levels of lipid significantly in this report (*P* > 0.05). We can partly explain this from the using of statins in the PCI essential treatment, whose effects on blood lipid may overwhelm the influence of DHI. The fluctuation of lipid in determination was also blamed for the result.

Oxidative stress leading to modification of LDL is a central paradigm of atherogenesis and plaque destabilization (Armstrong et al., 2006). DHI alleviated the oxidative stress via increasing SOD level and decreasing MDA content significantly (*P* < 0.00001). Cardiac function was also improved by DHI through increasing LVEF in patients (*P* = 0.006).

We have checked the methods used for the measurement of different biomarkers across the selected studies. All the indexes except NO were detected by the same or similar methods to keep the methodological consistency. NO was detected by nitrate reductase method in six trails (Xiao and Cao, 2010; Wang et al., 2011; Dong and Du, 2015; Guo J.F. et al., 2015; Liu et al., 2016; Zhang W.W. et al., 2016) whereas by ELISA in one study (Ye et al., 2017). No statistical significant was observed among individual studies, which mean the difference in methodological inconsistency didn’t add heterogeneity to the analysis of NO.

Acute coronary syndrome is a pathology characterized by complicated changes of inflammatory response, dyslipidemia, endothelium, platelet activation, oxidative stress, and cardiac functions et al. DHI exerts its pharmacological effects through a multi-components and targets way including anti-inflammatory, antioxidant, adjusting functions of vascular endothelial and cardiac, anti-agglutination of platelets to improve symptoms of ACS in patients after PCI. However, there are limitations to this research, such as the low quality of eligible trails, the lack of strict methodologies and the employed of sole race rather than a more varied population sample. It is necessary to examine the results using other rigorous and large-scale RCTs.

## Conclusion

These findings indicate that the combination of DHI and PCI significantly improve the TER and reduce incidence of MACE after PCI therapy. These effects are mediated by combined action of several mechanisms. In the present study, the combination could enhance the protection of vascular endothelial function through decreasing the ET-1 and vWF while increasing the level of NO and FMD. DHI combined with PCI plays an anti-inflammatory role by decreasing the level of IL-1, IL-6, IL-18, TNF-α, LpPLA2, MMP-9, pentraxin-3 and increasing level of IL-10 in serum. The combination displays an anticoagulation effect by decreasing level of CD62P, PAGT, PADT, FIB-C but not sP-sel in serum. It is likely that the combination also affects the blood lipid by regulating the contents of TC, TG, LDL, and HDL, but the results were not statistically significant. Cardiac function is improved by increasing LEVF but not LVED. The combination shows a regulation of oxidation state by decreasing content of MDA and increasing level of SOD. However, our findings must be handled with care because of the small sample size and low quality of clinic trials cited. Other rigorous and large-scale RCTs are in need to confirm these results.

## Author Contributions

J-BZ, X-FZ, and JW searched articles in electronic databases and wrote the manuscript. FW, J-XC, F-YY, and XS analyzed the data. J-BZ, YW, and Y-LL performed the data extraction. Y-JS designed the study and amended the paper.

## Conflict of Interest Statement

The authors declare that the research was conducted in the absence of any commercial or financial relationships that could be construed as a potential conflict of interest.
